# Supplementary material for: The C. elegans homolog of Sjögren’s Syndrome Nuclear Antigen 1 is required for the structural integrity of the centriole and bipolar mitotic spindle assembly
Source: bioRxiv. 2024 Oct 4:2024.10.03.616528. Preprint. [Version 1] doi: 10.1101/2024.10.03.616528 (PMC11722412; doi:10.1101/2024.10.03.616528)
Supplement: Supplement 1 [file media-1.pdf]

Table S1. *C. elegans* strains used in this study

N2	Wild type
EU3000	*sas-7(or1940[gfp::sas-7]) III; ItIs37 [pie-1p::mCherry::his-58 + unc-119(+)] IV*
OC210	*fem-1(hc17) IV*
OC779	*bsSi15 [pKO109: spd-2p-spd-2::mCherry::spd-2 3'-utr, unc-119(+)] I; bsSi30[pCW9: unc-119(+) pcdk-11.2::sfgfp::his-58::cdk-11.2 3' utr] II; unc-119(ed3) III*
OC786	*zyg-1 (bs212 [HA::zyg-1]) II*
OC908	*bsSi30[pCW9: unc-119(+) pcdk-11.2::sfgfp::his-58::cdk-11.2 3' utr] II; bsIs20[pNP99: unc-119(+) tbb-1p::mCherry::tbb-2::tbb-2 3'-utr]; bsIs2 [pCK5.5: Ppie-1::gfp::spd-2]*
OC953	*sas-6 (bs175 [sas-6::HA]) IV*
OC955	*sas-4 (bs177 [HA::sas-4]) III*
OC966	*sas-4 (bs186 [spot::sas-4]) III*
OC994	*sas-4(bs195 [sas-4::gfp]) III*
OC1002	*zyg-1 (bs197[zyg-1::spot]) II*
OC1013	*bsSi30[pCW9: unc-119(+) pcdk-11.2::sfgfp::his-58::cdk-11.2 3' utr] II; bsIs20[pNP99: unc-119(+) tbb-1p::mCherry::tbb-2::tbb-2 3'-utr]; bsIs2 [pCK5.5: Ppie-1::gfp::spd-2]; ssna-1 (bs182) / dpy-9 (tm9713) kvs-5 (tmIs1245)] IV*
OC1014	*ssna-1(bs182) / dpy9 (tm9713) kvs5 (tmIs1245) IV*
OC1018	*ssna-1 (bs206 [ssna-1::spot]) IV*
OC1020	*ssna-1(bs182)/ears-2(ve631[LoxP + myo-2p::gfp::unc-54 3' UTR + rps-27p::neoR::unc-54 3' UTR + LoxP]); fem1 (ht17ts) IV*
OC1021	*zyg-1 (bs197 [zyg-1::spot] II; ssna-1 (bs182) IV*
OC1040	*sas-4(bs195 [sas-4::gfp]) III; ssna-1 (bs206 [ssna-1::spot]) IV*
OC1046	*ssna-1(bs182)/dpy9 (tm9713) kvs5 (tmIs1245) IV*
OC1047	*zyg-1(it25) II*
OC1048	*zyg-1(it25) II; ssna-1(bs182)/dpy9 (tm9713) kvs5 (tmIs1245) IV*
OC1052	*ssna-1 (bs220 [1-99])/dpy9 (tm9713) kvs5 (tmIs1245) IV*
OC1068	*ssna-1 (bs222 [G7X])/dpy9 (tm9713) kvs5 (tmIs1245) IV*
OC1070	*ssna-1 (bs224 [Δ2-22])/dpy9 (tm9713) kvs5 (tmIs1245) IV*
OC1072	*ssna-1 (bs231 [Δ2-17])/dpy9 (tm9713) kvs5 (tmIs1245) IV*

OC1076	*ssna-1 (bs235 [Δ2-18])/dpy9 (tm9713) kvs5 (tmIs1245) IV*
OC1078	*ssna-1 (bs237[Δ2-18::spot])/dpy9 (tm9713) kvs5 (tmIs1245) IV*
OC1126	*spd-5(vie26[gfp::spd-5 +loxP]) I; ssna-1(bs206 [ssna-1::spot]) IV*
OC1129	*spd-5(vie26[gfp::spd-5 +loxP]) I; ltIs37[pAA64: unc-119(+) pie-1-mcherry-his58]*
OC1130	*spd-5(vie26[gfp::spd-5 +loxP]) I; ssna-1(bs182) IV; ltIs37[pAA64: unc-119(+) pie-1-mcherry-his58]*
OC1132	*ssna-1 (bs243[ssna-1::wrmScarlet) IV*
OC1134	*sas-6 (bs190 [sas-6::spot]) IV*
OC1135	*ssna-1(bs182), sas-6 (bs190 [sas-6::spot]) IV*
OC1138	*ssna-1(bs182)/ears-2(ve631[LoxP + myo-2p::gfp::unc-54 3' UTR + rps-27p::neoR::unc-54 3' UTR + LoxP]) IV*
OC1139	*ssna-1 (bs243 [ssna-1::wrmScarlet]) IV; ltSi560 [pPLG014; Pmex-5::gfp::his-11::tbb2_3'UTR, tbg-1::gfp::tbb-2_3'UTR; cb-unc-119(+)]V*
OC1166	*sas-1(t1476, bs272) III*
OC1177	*zyg-1(it25) II; bsSi15 [pKO109: spd-2p-spd-2::mCherry::spd-2 3'-utr, unc-119(+)] I; bsSi30[pCW9: unc-119(+) pcdk-11.2::sfgfp::his-58::cdk-11.2 3' utr] II*
OC1178	*zyg-1(it25) II; bsSi15 [pKO109: spd-2p-spd-2::mCherry::spd-2 3'-utr, unc-119(+)] I; bsSi30[pCW9: unc-119(+) pcdk-11.2::sfgfp::his-58::cdk-11.2 3' utr] II; ssna-1 (bs182)/ears-2(ve631[LoxP + myo-2p::GFP::unc-54 3' UTR + rps-27p::neoR::unc-54 3' UTR + LoxP]) IV*
OC1183	*bsSi15 [pKO109: spd-2p-spd-2::mCherry::spd-2 3'-utr, unc-119(+)] I; bsSi30[pCW9: unc-119(+) pcdk-11.2::sfgfp::his-58::cdk-11.2 3' utr] II; ssna-1 (bs182)/ears-2(ve631[LoxP + myo-2p::gfp::unc-54 3' UTR + rps-27p::neoR::unc-54 3' UTR + LoxP]) IV*
OC1205	*sas-5(bs199 [sas-5::spot]) V*
OC1207	*ssna-1(bs182)/ears-2(ve631[LoxP + myo-2p::gfp::unc-54 3' UTR + rps-27p::neoR::unc-54 3' UTR + LoxP]) IV; sas-5(bs199 [sas-5::spot]) V*
OC1209	*sas-7(or1940[gfp::sas-7]) III; ssna-1(bs182)/ears-2(ve631[LoxP + myo-2p::gfp::unc-54 3' UTR + rps-27p::neoR::unc-54 3' UTR + LoxP]), ItIs37 [pie-1p::mCherry::his-58 + unc-119(+)] IV*
OC1264	*sas-4 (bs186 [spot::SAS-4]) III; ssna-1(bs182)/ears-2(ve631[LoxP + myo-2p::gfp::unc-54 3' UTR + rps-27p::neoR::unc-54 3' UTR + LoxP]) IV*
OC1265	*sas-4(bs195 [sas-4::gfp]) III; ssna-1(bs182)/ears-2(ve631[LoxP + myo-2p::gfp::unc-54 3' UTR + rps-27p::neoR::unc-54 3' UTR + LoxP]) IV*

OC1335	*sas-1(t1476, bs272) III; ssna-1(bs182)/ears-2(ve631[LoxP + myo-2p::gfp::unc-54 3' UTR + rps-27p::neoR::unc-54 3' UTR + LoxP]) IV*
OC1344	*ssna-1 (bs356 [R18E::spot]) IV*

Table S2: crRNAs and repair templates used in this study

<table>
<tr><th>Allele</th><th>Background</th><th>crRNA (5’ -> 3’)</th><th>Repair template (5’ -> 3’)</th></tr>
<tr><td>bs199</td><td>N2</td><td>TTCGTGAAAAATACGCTCGC</td><td>CTGAACGAGAACGCCGTATTCGTGAAAAATACGCTCGC
AGAAAACCAGACCGTGTCCGTGCCGTCTCCCACTGGT
CCTCCTGATATCAAATGTGTTTAACTCTTGACGTTTTAAA</td></tr>
<tr><td>bs206</td><td>N2</td><td rowspan="8">CTTTGTGCGCAAAGAGTATC

GATATATTTACAGGCATTTT</td><td rowspan="5">GCAAAAGACGTTGGTGGACTTTGTGCGCAAAGAGTATC
AAGATACGAAACATCAGAAATATCCAGACCGTGTCCGTG
CCGTCTCCCACTGGTCCTCCTGAACTCTGAACAACTGTC
TCCCAAAAATGCCTGTAAATATATCAATTATCGACATAACTTC</td></tr>
<tr><td>bs233</td><td>bs231</td></tr>
<tr><td>bs237</td><td>bs235</td></tr>
<tr><td>bs249</td><td>bs217</td></tr>
<tr><td>bs357</td><td>bs286</td></tr>
<tr><td>bs218</td><td>N2</td><td>GCAAAAGACGTTGGTGGACTTTGTGCGCAAAGAGTATCAA
GATACGAAACATCAGAAATATGAACCGGAAGCGTGAACTCT
GAACAACTGTCTCCCAAAAATGCCTGTAAATATATCAATTATC
GACATAACTTC</td></tr>
<tr><td rowspan="2">bs242</td><td rowspan="2">N2</td><td>GCAAAAGACGTTGGTGGACTTTGTGCGCAAAGAGTATCAA
GATACGAAACATCAGAAATATGGATCCGCCGGATCCGCCG
CCGGATCCGGAGAGTTCGTCAGCAAGGGAGAGGCAGTTAT
CAAGGAGTTCATGCGTTTCAAGGTCCACATGGA</td></tr>
<tr><td>TATCAAGGAGTTCATGCGTTTCAAGGTCCACATGGAGGGAT
CCATGACCGAGGGACGTCACTCCACCGGAGGAATGGACGA
GCTCTACAAGTGAACTCTGAACAACTGTCTCCCAAAAATGCC
TGTAAATATATCAATTATCGACATAACTTC</td></tr>
<tr><td>bs243</td><td>bs242</td><td>CATGGAGGGATCCATGACCG</td><td>GTCAGCAAGGGAGAGGCAGTTATCAAGGAGTTCATGCGTTT
CAAGGTCCACATGGAGGGATCCATGAACGGACACGAGTTCG
AGATCGAGGGAGAGGGAGAGGGACGTCCATACGAGGGAAC
CCAAACCGCCAAGCTCAAGGTCACCAAGGGAGGACCACTC
CCATTCTCCTGGGACATCCTCTCCCCACAATTCATGTACGGA
TCCCGTGCCTTCACCAAGCACCCAGCCGACATCCCAGACTA
CTACAAGCAATCCTTCCCAGAGGGATTCAAGTGGGAGCGTG
TCATGAACTTCGAGGACGGAGGAGCCGTCACCGTCACCCAA
GACACCTCCCTCGAGGACGGAACCCTCATCTACAAGGTCAA
GCTCCGTGGAACCAACTTCCCACCAGACGGACCAGTCATGC
AAAAGAAGACCATGGGATGGGAGGCCTCCACCGAGCGTCTC
TACCCAGAGGACGGAGTCCTCAAGGGAGACATCAAGATGGC
CCTCCGTCTCAAGGACGGAGGACGTTACCTCGCCGACTTCA
AGACCACCTACAAGGCCAAGAAGCCAGTCCAAATGCCAGGA
GCCTACAACGTCGACCGTAAGCTCGACATCACCTCCCACAAC
GAGGACTACACCGTCGTCGAGCAATACGAGCGTTCCGAGGG
ACGTCACTCCACCGGAGGAATGGACGAGCTCTACAAG</td></tr>
<tr><td>bs182</td><td>N2</td><td>TAGAATCATGCATTTGCATT

CTTTGTGCGCAAAGAGTATC</td><td>TTCGTATTTGAACAATTACTGACTAATTTCCTCCGAATGCAAA
TGCATGATTCTAGAACAAAAAAAACATCAGAAATATTGAACTC
TGAACAACTGTCTC</td></tr>
</table>

bs215	N2	CTGTGAGACGGCGTTCCTCT	GAAGATAAACATTTATAGTAATATTTCAGACATCCAAGCGCTCA GAGAGGAACGCCGTCTCACAGAATCGTCGATTCGAAAAATG
bs216	bs215	AGACATCCAAGCGCTCGCGG	GTAAGAGCAAATTGAAGATAAACATTTATAGTAATATTTCAGAC ATCCAAGCGCTCGCGGAAGAACGCCGTCTCACAGAATCGTC GATTCGAAAAATGGA
bs217			GTAAGAGCAAATTGAAGATAAACATTTATAGTAATATTTCAGAC ATCCAAGCGCTCGCGGCAGCACGCCGTCTCACAGAATCGTC GATTCGAAAAATGGAAA
bs220	N2	CTTTGTGCGCAAAGAGTATC	GCAAAAGACGTTGGTGGACTTTGTGCGCAAAGAGTATCAAGA TTGAACTCTGAACAACTGTCTCCCAAAAATGCCTGT
bs222	N2	AAAATGTCTTCTCGATCTAC	TGCATGATTCTAGAACAAAAAATGTCTTCTCGATCTACATGAA GCTTTGATGAAATATCACAGTGTAAGAGCAAATT
bs224	bs215	AAATGTCTTCTCGATCTAC AGACATCCAACGTCTCCGAG	CCGAATGCAAATGCATGATTCTAGAACAAAAAATGCGCCGTC TCACAGAATCGTCGATTCGAAAAATGGA
bs231	N2	AAAATGTCTTCTCGATCTAC CTGTGAGACGGCGTTCCTCT	CCGAATGCAAATGCATGATTCTAGAACAAAAAATGCGTCTC AGAGAGGAACGCCGTCTCACAGAATCGTCGATTCGAAAAA
bs235	N2		CCGAATGCAAATGCATGATTCTAGAACAAAAAATGCTCAG AGAGGAACGCCGTCTCACAGAATCGTCGATTCGAAAAA
bs284	N2		TGCATGATTCTAGAACAAAAAATGTCTTCTCGAAGCACAG GAAGCTTTGATGAAATATCACAGGGTAAGAGCAAATTGAA GATAAACATTTATAGTAATATTTCAGAGATCCAACGTCTCAG AGAGGAACGCCGTCTCACAGAATCGTCGATTCGAAAAA
bs286	N2	CTGTGAGACGGCGTTCCTCT	GAAGATAAACATTTATAGTAATATTTCAGACATCCAAGAGCTCA GAGAGGAACGCCGTCTCACAGAATCGTCGATTCGAAAAATG
bs312	bs284	CTTTGTGCGCAAAGAGTATC	CGCAAAAGACGTTGGTGGACTTTGTGCGCAAAGAGGAGCAG GATACGAAACATCAGAAATATTGAACTCTGAACAAC
bs314	bs286		
bs335	N2		

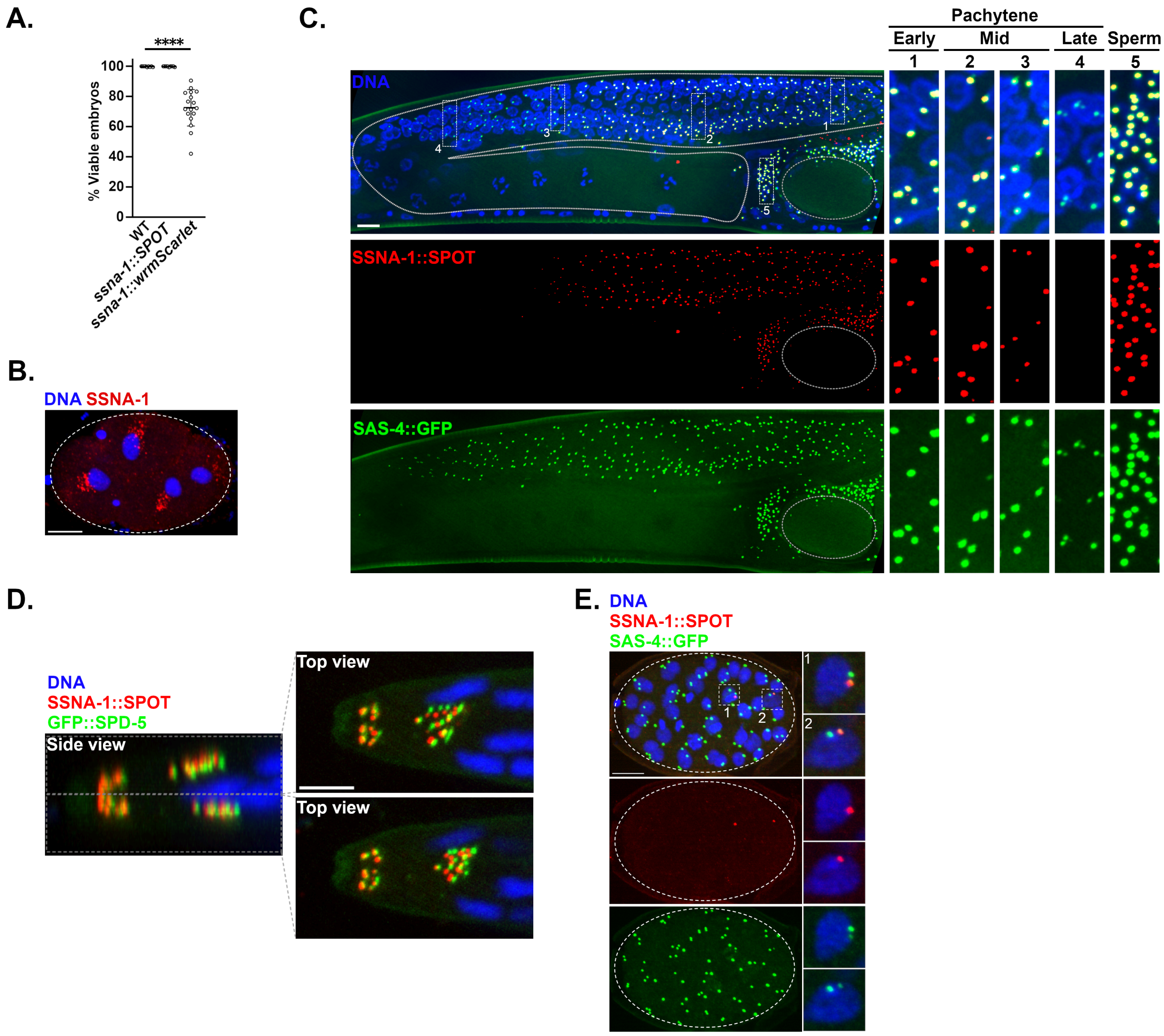

Fig. S1: SSNA-1 is a stable component of centrioles with broad distribution in the worm. A.

Quantification of embryonic viability of strains expressing different C-terminal epitope tagged versions of endogenous SSNA-1. Each datapoint represent progeny of a single hermaphrodite. **B.** Representative image of an embryo stained for endogenous SSNA-1 and DNA. Bar = 10 µm. **C.** Localization of SSNA-1::SPOT in the germline with SAS-4::GFP marking centrioles, showing that SSNA-1 is present on centrioles on immature female germ cells as well as sperm. SSNA-1 is lost from germ cell centrioles during mid-pachytene (box 3), prior to SAS-4 (box 4). Bar = 10 µm. **D.** SSNA-1::SPOT is found adjacent to the acentriolar centrosome (marked with GFP::SPD-5) in amphid neurons of L1 larvae. Bar = 10 µm. **E**. An embryo produced by mating SSNA-1::SPOT males to SAS-4::GFP hermaphrodites stained for SAS-4::GFP (green), SSNA-1::SPOT (red) and DNA (blue). Even after many cell cycles SSNA-1::SPOT remains associated with the two original sperm centrioles revealing that SSNA-1 is a stable component of centrioles. Scale bar = 10 µm.

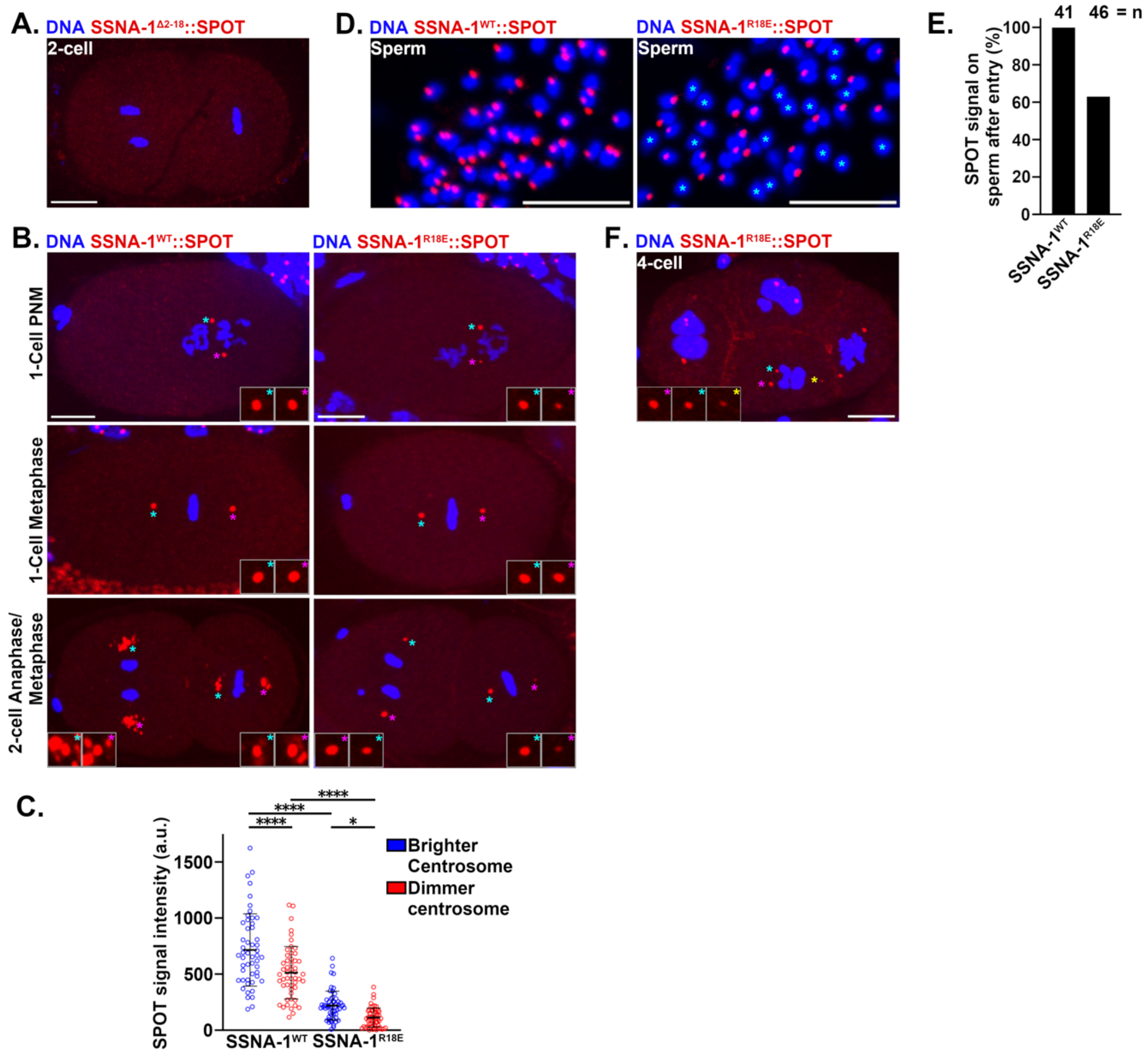

Fig. S2: SSNA-1 oligomerization and MT binding are required for centriolar and satellite localization. A. Representative image of a two-cell stage embryo expressing SSNA-1$^{\Delta 2\text{-}18}$::SPOT showing that SSNA-1 fails to localize to the centriole and satellite-like structures. Scale bar = 10 µm. **B.** Localization of SSNA-1^{WT}::SPOT (left column) and SSNA-1^{R18E}::SPOT (right column)

from pronuclear migration (PNM) to the late 2-cell stage in strains grown at 20°C. Full-size images are maximum intensity projections while insets are 2X magnifications of the center z plane of each centrosome indicated by colored asterisks. Scale bar = 10 µm. **C.** Quantification of SSNA-1 signal intensity from B showing that the centriole levels of SSNA-1^{R18E}::SPOT are strongly reduced relative to wild-type. Also notice that in both the wild-type and mutant, that one centrosome is brighter than the other. **D.** Maximum intensity projections of sperm from SSNA-1^{WT}::SPOT and SSNA-1^{R18E}::SPOT strains. Note that in the wild type, all sperm nuclei are associated with a SSNA-1::SPOT focus while many sperm in the SSNA-1^{R18E}::SPOT strain lack a focus (asterisks). Scale bar = 10 µm. **E.** Quantitation showing the percentage of wild-type and R18E mutant sperm that possess a focus of SSNA-1. **F.** A four-cell stage embryo expressing SSNA-1^{R18E}::SPOT grown at 25°C. Note the presence of a multipolar spindle in the EMS cell at bottom. Full-scale images are maximum intensity projections while insets are 2X magnifications of the center z plane of each centrosome as marked by colored asterisks. Scale bar = 10µm.

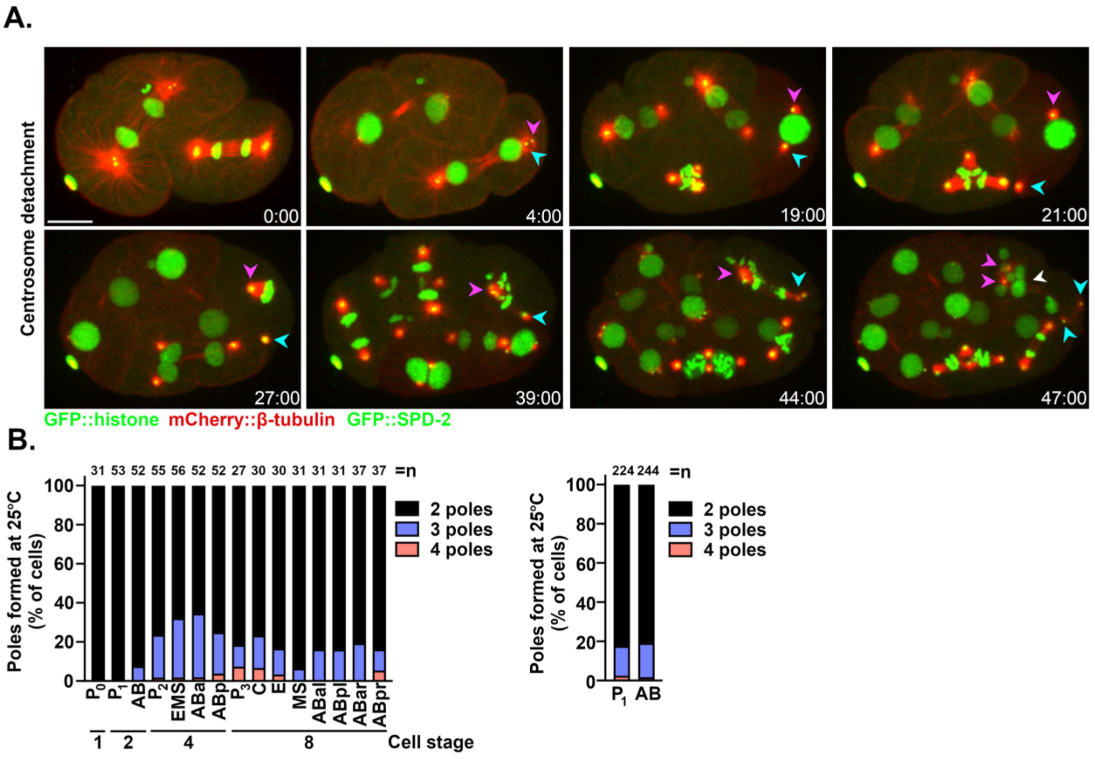

Fig. S3: Cell division defects during development of *ssna-1(Δ)* embryos. A. Frames from a time-lapse recording of an *ssna-1(Δ)* embryo expressing GFP::SPD-2 (green); GFP::histone (green); and mCherry::β-tubulin (red). Magenta and cyan arrowheads indicate the two centrosomes that arise from one pole of the P_1 spindle (t=4:00). One centrosome stays associated with the P_2 nucleus (magenta) while the other (cyan) migrates to the cell periphery (t=21:00). The itinerant centrosome makes a late contribution to bipolar spindle assembly (t=44:00), but

chromosome segregation defects lead to micronuclei formation (white arrowhead) (t=47:00). Note that both centrosomes duplicated. Scale bar = 10 μm. **B.** Distribution of multipolar spindle formation among the early embryonic blastomeres (n= number of cells scored). **C.** Percentage of cells within the P_1 and AB lineages that form multipolar spindles.

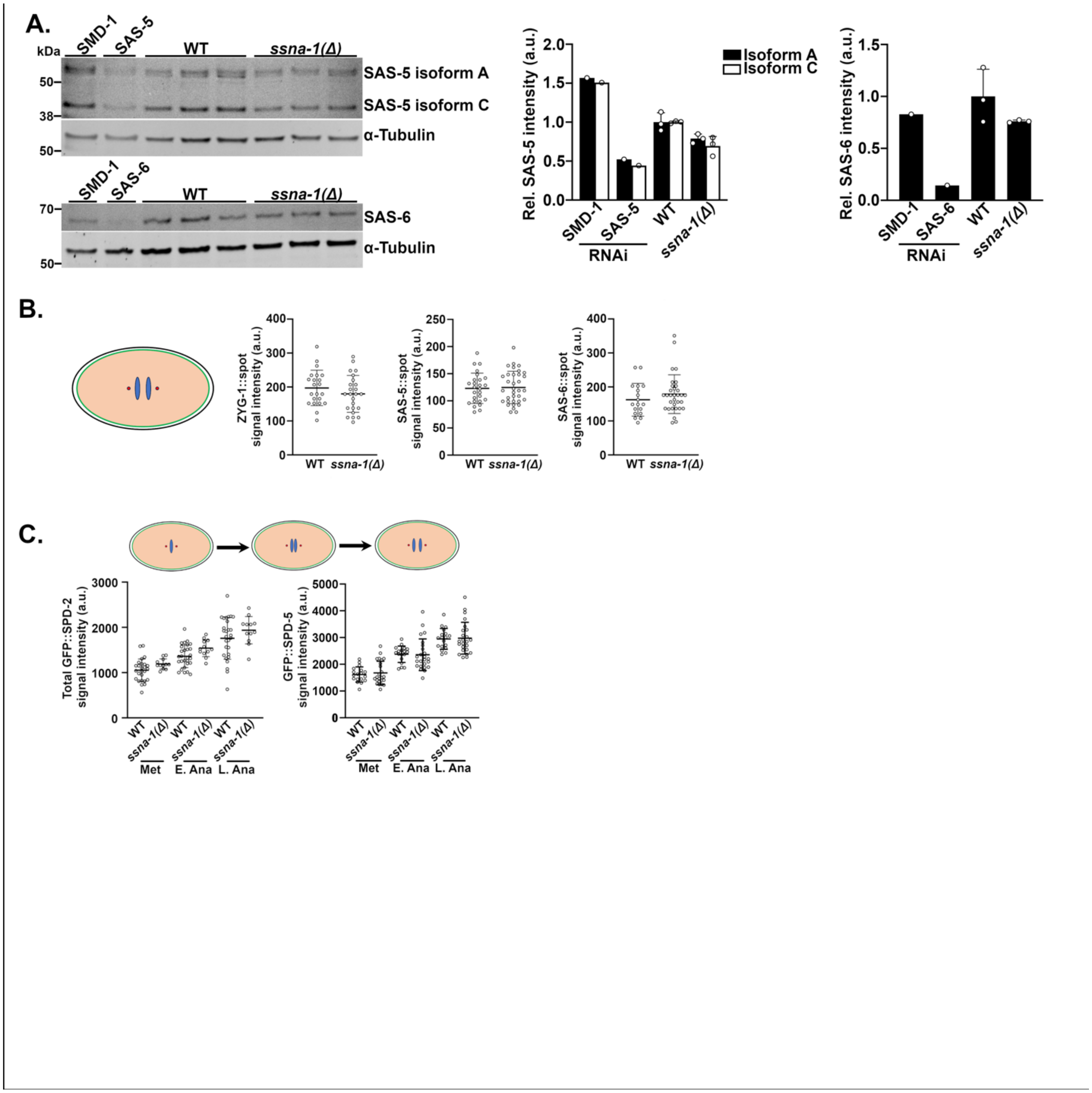

Fig. S4. Loss of SSNA-1 is not associated with overexpression of centriole duplication factors. A. Immunoblots of SAS-5 and SAS-6 levels in extracts prepared from gravid hermaphrodites (left). Quantitation of blots (right) shows the relative levels after normalization to α-tubulin. **B.** Centriole-associated levels of ZYG-1::SPOT, SAS-5::SPOT, and SAS-6::SPOT at anaphase in the zygote. **C**. Centrosome-associated levels of GFP::SPD-2 (left) and GFP::SPD-5 (right) from metaphase through anaphase in the zygote, as measured by quantitative

immunofluorescence. In panels B and C, all differences between wild-type and *ssna-1(Δ)* mutants are nonsignificant.

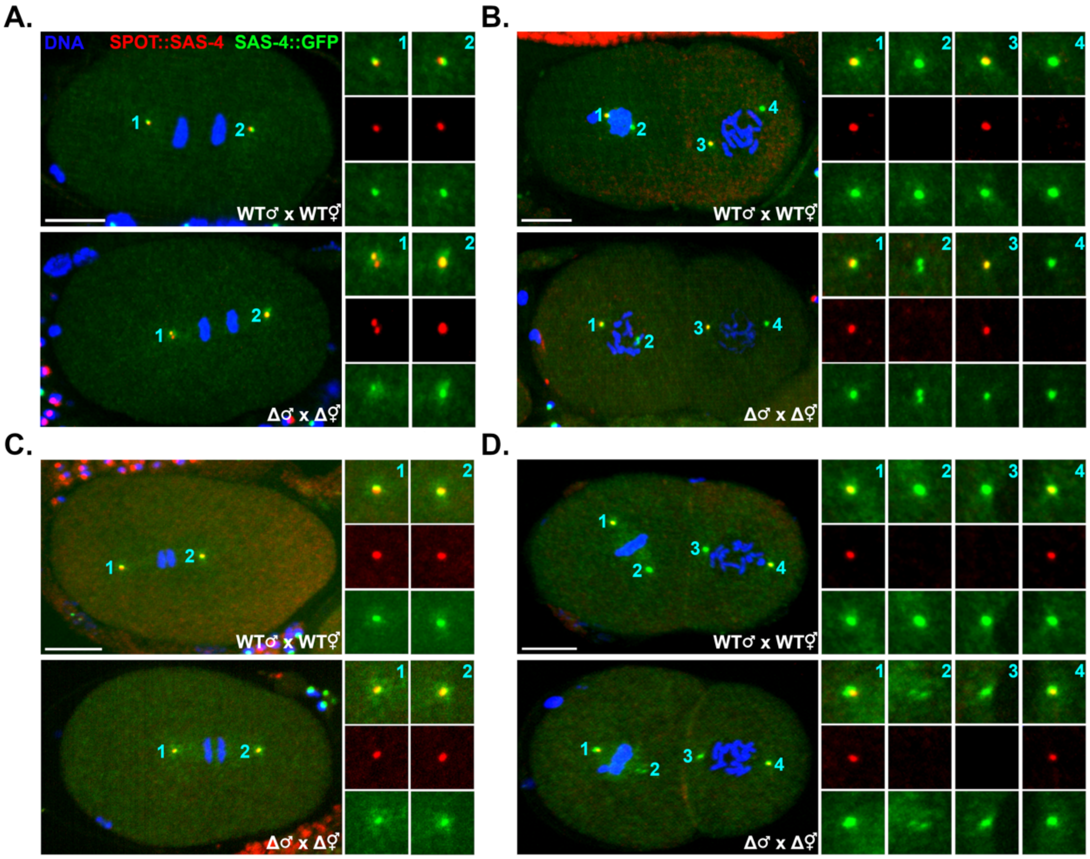

Fig S5. Loss of *ssna-1* results in centriole fragmentation rather than premature disengagement. A-D. Representative images of embryos produced from *SPOT::sas-4* male x *sas-4::GFP* hermaphrodite matings. Insets are 2X magnifications. All images are maximum intensity projections. Scale bars = 10 μm**. A.** WT and *ssna-1(Δ)* embryos at mid anaphase. In WT embryos, mother (red) and daughter (green) centrioles remain associated prior to normal disengagement in late anaphase/telophase while in *ssna-1(Δ)* embryos, mother (red) centrioles

fracture (centrosome 1). One of these two red centriole fragments remains associated with the green daughter centriole indicating the centriole pair is still engaged. **B.** WT and *ssna-1(Δ)* embryos during prophase and prometaphase. Note that all red centrioles and their green daughters are coincident and therefore engaged. Note also an instance of apparent centriole fragmentation of a green centriole in the *ssna-1(Δ)* embryo (centrosome 2). **C and D.** Representative images of one- (C) and two-cell (D) embryos showing that in both wild-type and *ssna-1(Δ)* embryos, all red paternally derived centrioles remain engaged with their gene daughters through early anaphase (WT = 64 centrosomes and *ssna-1(Δ)* = 56 centrosomes).

Movie S1. SSNA-1 satellite-like structures display dynamic behavior. Time-lapse video corresponding to Fig. 2E of an embryo expressing SSNA-1::wrmScarlet, GFP::histone, and γ-tubulin::GFP. A side view of the ABpl blastomere dividing is shown. SSNA-1 satellites display dynamic behavior during division whereby they disperse at the end of mitosis following PCM breakdown and spread out across the nucleus. As the cell progresses through mitosis, the satellites accumulate around the centrosome of each pole. Maximum accumulation is achieved by late metaphase/early anaphase.
